# Sex-related differences in sleep slow wave activity in major depressive disorder: a high-density EEG investigation

**DOI:** 10.1186/1471-244X-12-146

**Published:** 2012-09-18

**Authors:** David T Plante, Eric C Landsness, Michael J Peterson, Michael R Goldstein, Brady A Riedner, Timothy Wanger, Jeffrey J Guokas, Giulio Tononi, Ruth M Benca

**Affiliations:** 1 Department of Psychiatry, University of Wisconsin School of Medicine and Public Health, Madison, WI, USA

## Abstract

**Background:**

Sleep disturbance plays an important role in major depressive disorder (MDD). Prior investigations have demonstrated that slow wave activity (SWA) during sleep is altered in MDD; however, results have not been consistent across studies, which may be due in part to sex-related differences in SWA and/or limited spatial resolution of spectral analyses. This study sought to characterize SWA in MDD utilizing high-density electroencephalography (hdEEG) to examine the topography of SWA across the cortex in MDD, as well as sex-related variation in SWA topography in the disorder.

**Methods:**

All-night recordings with 256 channel hdEEG were collected in 30 unipolar MDD subjects (19 women) and 30 age and sex-matched control subjects. Spectral analyses of SWA were performed to determine group differences. SWA was compared between MDD and controls, including analyses stratified by sex, using statistical non-parametric mapping to correct for multiple comparisons of topographic data.

**Results:**

As a group, MDD subjects demonstrated significant increases in all-night SWA primarily in bilateral prefrontal channels. When stratified by sex, MDD women demonstrated global increases in SWA relative to age-matched controls that were most consistent in bilateral prefrontal regions; however, MDD men showed no significant differences relative to age-matched controls. Further analyses demonstrated increased SWA in MDD women was most prominent in the first portion of the night.

**Conclusions:**

Women, but not men with MDD demonstrate significant increases in SWA in multiple cortical areas relative to control subjects. Further research is warranted to investigate the role of SWA in MDD, and to clarify how increased SWA in women with MDD is related to the pathophysiology of the disorder.

## Background

Sleep disturbance has diagnostic, clinical, and functional significance in major depressive disorder (MDD). Self-report of insomnia and/or hypersomnia is a diagnostic criterion for MDD, and sleep-related complaints occur in the majority of MDD patients [1,2]. Sleep-related clinical complaints are important in the natural history of the disorder, as they increase the risk of developing a depressive episode [3-7], attempting suicide [8,9], and relapsing after remission [10-12]. Additionally, myriad studies have examined polysomnographic measures in MDD, demonstrating alterations in sleep continuity, rapid eye movement (REM) sleep, and slow wave sleep (SWS) [13,14]. Scoring of SWS relies on visual assessment of frequency, amplitude, and proportion of delta waves [15,16], which can occur in all stages of NREM sleep and are not exclusive to SWS. Slow wave activity (SWA), which represents the power density in the 1-4.5Hz range in all stages of NREM sleep, more accurately captures the variation in slow oscillations during sleep that is not reflected by traditional sleep staging [17,18].

SWA has been established as a marker of sleep homeostasis, as it increases in proportion to prior wakefulness and declines with sleep [19,20]. Based in part on the antidepressant effects of sleep deprivation, it has been hypothesized that MDD involves deficiency in sleep homeostatic processes—the S-deficiency hypothesis of depression [21,22]. Prior studies that have examined SWA in MDD using spectral analysis support this hypothesis, particularly suggesting decrements of SWA in the first portion of the night [23,24]. However, findings have not been consistent across studies, with other investigations failing to demonstrate significant differences in SWA in depressed subjects relative to healthy controls [25-28]. Moreover, significant sex-related effects on SWA in MDD have been described, with increases in SWA among depressed women and decreases in SWA among depressed men, relative both to each other and to healthy comparison subjects [29-31].

A major limitation of prior studies of SWA in MDD is the use of limited central derivations of the electroencephalogram (EEG) to quantify SWA. Since SWA is most prominent during sleep in frontal brain regions [32-35], the use of central channels to assess SWA may not optimally reflect differences in sleep homeostatic processes in depression. Furthermore, the use of a single or only a few EEG derivations to perform spectral analysis provides limited spatial resolution, and thus, topographic alterations in SWA would not be evident using these approaches. Therefore, this study was conducted using high-density (hd) EEG to evaluate the topography of sleep SWA in MDD relative to healthy age and sex-matched comparison subjects. We hypothesized that differences in SWA between MDD and control subjects would be most pronounced in frontal channels, and that there would be differential effects of sex on SWA in MDD subjects, with women with MDD demonstrating increases and men with MDD demonstrating reductions in SWA compared to age and sex-matched healthy comparison subjects.

## Methods

### Subjects

Thirty right-handed outpatient MDD subjects (19 female) were selected from a larger study on sleep homeostasis in neuropsychiatric disorders, conducted at the University of Wisconsin-Madison. MDD was diagnosed via the Structured Clinical Interview for DSM-IV Axis I disorders (SCID) [36] and global depression severity was evaluated with the clinician-administered 17-item Hamilton Rating Scale for Depression (HRSD) [37]. For inclusion, subjects were required to have at least moderate depression defined as HRSD > 15 and be free of psychotropic medications (or other agents that could alter sleep architecture) for ≥1 month. In addition, subjects were unipolar and had no history of psychosis or active drug/alcohol dependence. Subjects were free of significant neurological and medical conditions, including evidence of sleep disordered breathing and sleep related movement disorders, verified by in-laboratory polysomnography (see below). Age and sex-matched healthy comparison subjects were evaluated with the non-patient SCID [38] to rule out current or past psychiatric disorders.

All subjects provided informed consent and were instructed to maintain regular sleep-wake schedules, avoid napping, and to limit the use of caffeinated or alcoholic beverages for the duration of the study. Adherence was monitored using sleep-diaries and wrist motor actigraphy (Actiwatch, Mini-Mitter, Bend, OR). This study was approved by the Institutional Review Board of the University of Wisconsin-Madison.

### Study design

All subjects underwent in-laboratory hdEEG polysomnography (PSG) that utilized 256 channel hdEEG (Electrical Geodesics Inc., Eugene, OR), as well as standard monitoring with electrooculogram (EOG), sub-mental electromyogram (EMG), electrocardiogram (ECG), bilateral tibial EMG, respiratory inductance plethysmography, pulse oximetry, and a position sensor. Participants arrived at the laboratory between 20:00 and 21:00 for set-up that took approximately two hours, and then were allowed to sleep undisturbed in the laboratory beginning within one hour of their usual bedtime. Sleep hdEEG recordings were collected with vertex-referencing, using NetStation software (Electrical Geodesics Inc., Eugene, OR).

### Spectral analysis

HdEEG signals were sampled at 500 Hz, first-order high-pass filtered in NetStation (0.1Hz), downsampled to 128 Hz, band-pass filtered (2-way least-squares FIR, 1-40 Hz) in MATLAB (The MathWorks Inc., Natick, MA), and average-referenced to the average scalp voltage computed in all channels. Semi-automatic artifact rejection was conducted to remove channels with high-frequency noise or interrupted contact with the scalp during individual epochs. Channels for which artifact affected the majority of the recording were excluded. Spectral analysis of NREM sleep was performed for each channel in consecutive 6-second epochs (Welch’s averaged modified periodgram with a Hamming window). To increase the signal-to-noise ratio, analyses were restricted to the 185 channels overlaying the scalp [39]. Sleep staging was performed by a registered polysomnographic technologist in 30-second epochs according to standard criteria [16] using Alice® Sleepware (Philips Respironics, Murrysville, PA) based on 6 EEG channels at approximate 10-20 locations (F3, F4, C3, C4, O1, and O2) re-referenced to the mastoids, sub-mental EMG and EOG.

### Slow wave activity time course

SWA time course was quantified using similar methods described by other groups [29,40]. The exponential decay function utilized was as follows: SWA_*t*_ = (SWA_0_ * e^-*rt*^) + SWA_∞_, in which SWA_*t*_ is the SWA value at a given time point *t*, SWA_0_ represents the hypothetical SWA value at time 0, *t* indicates time (min) from sleep onset, *r* is the rate (min^-1^) of exponential decay, and SWA_∞_ indicates the SWA value of the decay function’s asymptote. To optimize fit for each decay function, for each subject, relative SWA for each NREM period was calculated as a percent of the average SWA across the first NREM periods 1-4, and NREM period midpoints were used to derive the corresponding time point values. NREM periods were defined using duration and endpoint criteria in a manner similar to prior studies [41], with the exception that NREM periods were inclusive of all stages of NREM sleep (N1-N3).

### Statistics

Differences in all-night SWA (both global average of 185 channels and channel-by-channel) between MDD and matched controls were examined using 2-tailed, unpaired t-tests. Given aforementioned sex-related differences in SWA in MDD [29,30], as well as reported differences in SWA between healthy men and women that vary by age [42-44], additional analyses between MDD and controls stratified by sex were performed to maintain age-matching of subjects. Also, effects of NREM period were explored given the known decline of SWA across NREM periods and prior studies that have demonstrated decrements in SWA in MDD primarily occurring in the first NREM period [24]. To correct for multiple comparisons of the topographical hdEEG data, statistical non-parametric mapping with suprathreshold cluster tests was utilized [45]. For correlational analyses of SWA with polysomnographic and clinical data, hdEEG sleep data were first cleaned for outliers using a threshold of ± 2.5 standard deviations from the mean at each channel. Statistical relationship between variables and SWA in each channel was assessed using linear regression. To limit spurious findings due to multiple comparisons, correlation analyses of PSG variables were limited to those measures significantly different between groups. Additionally, topographic correlations were considered significant only if clusters of ≥3 contiguous channels were significant at α=0.05 within cortical regions of significant difference between groups. Statistical analyses were performed using MATLAB (The MathWorks Inc., Natick, MA) and STATISTICA (StatSoft Inc., Tulsa, OK).

## Results

MDD subjects were young to middle aged (mean 26.0 ± 9.1; range 18 to 53 years) with moderate depression (mean HRSD 18.6 ± 2.7; range 15 to 25) and had not taken psychotropic medications within ≥ 5 months of enrollment (Table 1). Eleven MDD participants reported at least one prior depressive episode. HC subjects did not report a personal or family history of mood disorders. Polysomnography demonstrated significant differences between groups, with MDD subjects demonstrating decreased total sleep time (TST) and increased arousal index (AI) (Table 1). When stratified by sex, there were no significant differences in polysomnographic variables between women with MDD and female controls, but depressed men had significantly higher AI and lower sleep efficiency (SE) than male controls (Table 1).

**Table 1:** Demographic, clinical, and polysomnographic data

	**MDD**	**HC**	**p**	**MDD Females**	**HC Females**	**p**	**MDD Males**	**HC Males**	**p**
	**(*N*** **= 30)**	**(*N*** **= 30)**		**(*N*** **= 19)**	**(*N*** **= 19)**		**(*N*** **= 11)**	**(*N*** **= 11)**	
Age (years)	26.0 (9.1)	25.4 (8.5)	.793	23.4 (5.6)	23.1 (6.2)	.891	30.6 (12.1)	29.4 (10.7)	.811
HRSD-17	18.6 (2.7)	--		19.0 (3.0)	--		17.9 (1.6)	--	
TST (min.)	377.8 (66.3)	412.7 (47.5)	.023	390.5 (54.0)	419.6 (49.8)	.093	356.0 (81.7)	400.8 (42.9)	.122
WASO (min.)	43.3 (30.2)	30.2 (20.7)	.055	38.1 (26.4)	30.7 (20.6)	.345	52.4 (35.4)	29.4 (21.8)	.082
AI (#/hr.)	10.6 (5.6)	7.9 (4.3)	.043	9.4 (5.5)	8.2 (3.8)	.424	12.6 (5.5)	7.4 (5.3)	.036
SE (%)	86.5 (7.9)	89.6 (7.4)	.132	88.7 (7.1)	89.2 (8.6)	.846	82.8 (8.1)	90.1 (4.9)	.018
SOL (min.)	15.5 (16.5)	18.6 (24.3)	.566	11.6 (10.5)	20.6 (28.0)	.199	22.1 (22.7)	15.1 (16.7)	.414
N1 (%)	8.1 (5.3)	7.9 (4.7)	.926	7.5 (5.5)	7.7 (5.0)	.897	9.0 (5.0)	8.3 (4.2)	.724
N2 (%)	57.7 (8.1)	60.4 (6.1)	.151	56.6 (8.6)	60.4 (5.2)	.111	59.5 (7.2)	60.4 (7.8)	.788
N3 (%)	16.8 (9.2)	14.8 (7.1)	.348	20.0 (7.6)	15.9 (6.3)	.075	11.2 (9.3)	13.0 (8.2)	.651
REM (%)	18.1 (6.6)	16.9 (5.0)	.448	15.9 (6.5)	16.0 (5.2)	.923	22.3 (4.8)	18.4 (4.5)	.071
REML (min.)	131.2 (77.4)	122.2 (69.7)	.641	158.1 (78.4)	131.1 (71.2)	.275	80.1 (43.7)	106.7 (67.4)	.302
SWA (μV^2^/Hz)	22.7 (13.1)	17.3 (6.8)	.054	28.8 (12.4)	19.3 (6.8)	.006	12.1 (5.4)	13.9 (5.3)	.442

*MDD*, major depressive disorder; *HC*, healthy control; *HRSD-17*, Hamilton Rating Scale for Depression (17-item); *TST*, total sleep time; *WASO*, wake after sleep onset; *AI*, arousal index; *SE*, sleep efficiency (TST/time in bed); *SOL*, sleep onset latency; *N1/2/3*, NREM stage 1/2/3 (% of TST); *REM*, stage REM (% of TST); *REML*, REM latency (time from sleep onset to first REM sleep epoch); *SWA*, global slow wave activity (EEG power in 1 – 4.5 Hz range average 185 channels);

Values are displayed as mean (standard deviation).

*2-tailed, independent samples *t*-tests.

**Figure 1:**
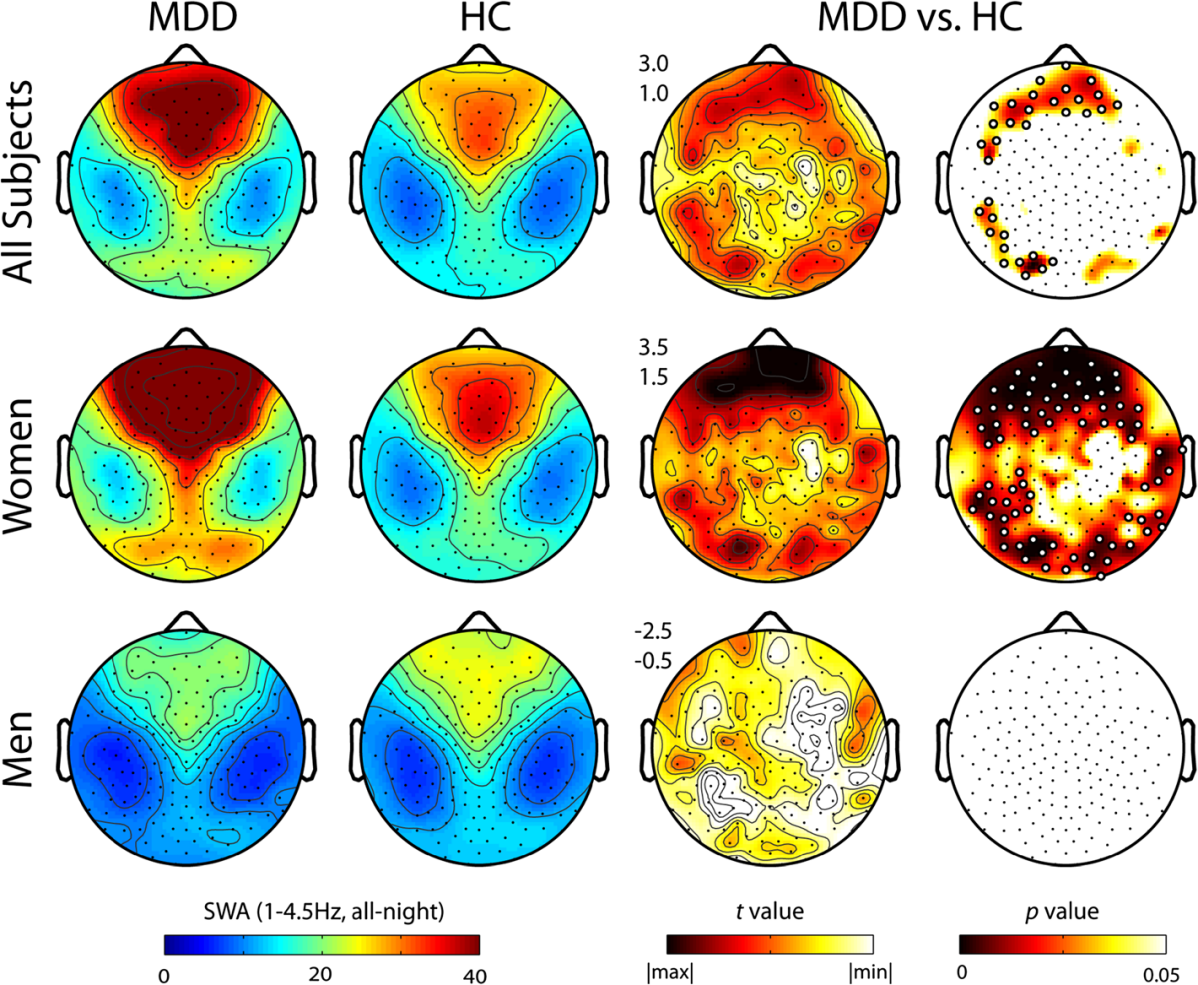
**Topographic all-night SWA (1-4.5 Hz) in MDD subjects versus healthy controls, both unstratified and stratified by sex.** T-values plotted for the comparisons between groups (2-tailed, unpaired *t*-test) at each channel. The minimum and maximum t-values for each map are plotted in white and black respectively, with the corresponding numeric range for color scale (upper left). Corresponding p-values plotted for each channel with white dots denoting channels with significant between-group differences following statistical non-parametric mapping with suprathreshold cluster tests to correct for multiple comparisons.

All-night global SWA (average of 185 channels) was not significantly different between MDD and HC groups; however, there was a trend towards increased global SWA in MDD (Table 1). Topographic analysis demonstrated significantly increased SWA in MDD relative to HC, primarily in bilateral prefrontal channels, as well as left lateral parietal and occipital channels (Figure 1).

When groups were stratified by sex, female MDD subjects demonstrated significantly higher global all-night SWA compared to age-matched female controls (28.8 μV^2^/Hz ± 12.4 vs. 19.3 μV^2^/Hz ± 6.8, p = 0.006). Topographic analysis demonstrated significant increases in SWA in MDD women relative to HC women that were most consistent in bilateral prefrontal channels, and additionally significant in multiple other cortical regions including frontal, lateral parietal, and occipital regions (Figure 1). However, neither global (12.1μV^2^/Hz ± 5.4 vs. 13.9μV^2^/Hz ± 5.3, p = 0.44) nor topographic differences were observed for MDD men relative to age-matched male controls (Figure 1).

**Figure 2:**
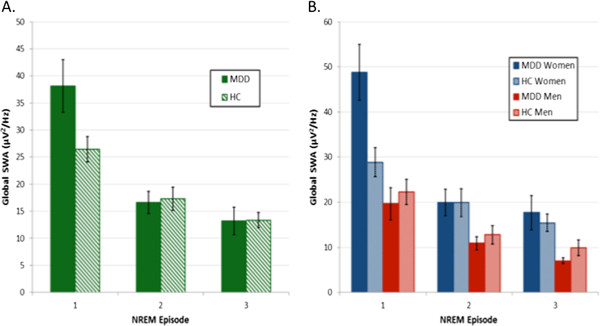
**Global SWA (1-4.5Hz) across NREM periods for MDD and healthy controls A) unstratified and B) stratified by sex.** Error bars represent standard error of the mean.

Given the sex-related differences in all-night global SWA observed, analyses both unstratified and stratified by sex were performed to examine the role of NREM period. In the unstratified analysis, a 2 (group) by 3 (NREM period) mixed model ANOVA was utilized, which showed a main effect of NREM period (F_2,46_ = 39.28, p < 0.0001) and a significant group x period interaction (F_2,92_ = 4.83, p = 0.01), without main effect of group (F_1,46_ = 0.81,; p = 0.37). Post hoc analyses, using Tukey’s HSD to correct for multiple comparisons, showed a significant decline of global SWA from NREM1 to NREM3 for both MDD (p = 0.0001) and HC (p = 0.001), with no significant differences between MDD and HC groups in any NREM period (Figure 2). In the stratified analyses, a 4 (group) by 3 (NREM period) mixed model ANOVA was utilized to examine global SWA across the night among groups stratified by sex. ANOVA demonstrated a main effect of period (F_2,44_ = 40.06, p < 0.0001) and a group x period interaction (F_6,88_ = 5.76, p < 0.0001). Post hoc analysis demonstrated a significant decline of global SWA from NREM1 to NREM3 for MDD (p = 0.0001) and HC women (p = 0.012), but not for MDD or HC men after correcting for multiple comparisons (Figure 2). MDD women showed a trend towards greater global SWA than age-matched HC women (p = 0.097) in NREM1, however, there were no other differences between MDD subjects of either sex and age-matched HC subjects in NREM2 or NREM3.

Subsequent topographic analysis of SWA in NREM1 demonstrated nearly identical patterns as all-night SWA in both stratified and unstratified analyses (See Additional file 1: Figure S1). Additional exploratory topographic analyses of SWA in NREM2 and NREM3 demonstrated no significant differences between groups (either stratified or unstratified by sex).

**Figure 3:**
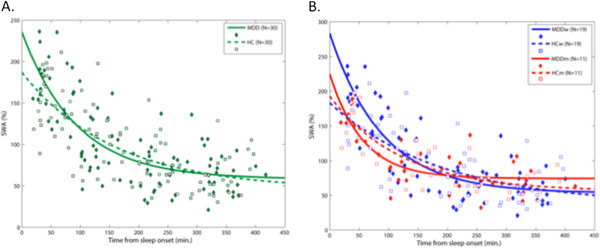
**Time course of relative slow wave activity (SWA) across non-rapid eye movement (NREM) sleep periods.** Individual SWA values per NREM sleep period (expressed as a percentage of all night SWA) are plotted at period midpoint relative to sleep onset for MDD and healthy controls **A**) unstratified and **B**) stratified by sex. All data from the first four NREM periods were included to maximize fit of the exponential function. Lines represent exponential functions that were fitted to the data using the equation SWA_*t*_ = (SWA_0_ * e^-*rt*^) + SWA_∞_, in which SWA_*t*_ is the SWA value at a given time point *t*, SWA_0_ represents the hypothetical SWA value at time 0, *t* indicates time (min) from sleep onset, *r* is the rate (min^-1^) of exponential decay, and SWA_∞_ indicates the SWA value of the decay function’s asymptote.

Fitted exponential decay functions are depicted in Figure 3 and calculated SWA time course variables presented in Table 2. In the unstratified analysis, there was a trend for greater SWA_0_ in MDD relative to control subjects (p = 0.10). When SWA time course was stratified by sex, MDD women demonstrated significantly greater SWA_0_ relative to HC women (p = 0.03). There were no other significant differences in SWA time course variables in either unstratified or stratified analyses.

**Table 2:** SWA time course variables derived from fitted exponential decay functions*

	**MDD**	**HC**		**MDD Females**	**HC Females**		**MDD Males**	**HC Males**	
	**(*N*= 30)**	**(*N*= 30)**	**p**	**(*N*= 19)**	**(*N*= 19)**	**p**	**(*N*= 11)**	**(*N*= 11)**	**p**
R^2^	0.63	0.55	--	0.71	0.51	--	0.66	0.65	--
SWA_0_	177.34	141.00	0.10	230.04	146.86	0.03	150.79	137.30	0.68
(CI)	(143.41-211.27)	(113.44-168.55)		(176.43-283.66)	(95.57-198.15)		(89.50-212.09)	(102.63-171.97)	
*r*	0.0108	0.0063	0.18	0.0112	0.0053	0.15	0.0178	0.0082	0.16
(CI)	(0.0060-0.155)	(0.0017-0.0110)		(0.0060-0.0163)	(−0.0011-0.0116)		(0.0057-0.0299)	(0.0011-0.0154)	
SWA_∞_	58.30	45.82	0.52	53.24	36.49	0.63	74.35	55.74	0.28
(CI)	(41.86-74.74)	(10.23-81.40)		(33.41-73.08)	(−33.48-106.47)		(60.13-88.56)	(22.47-89.02)	

*Values derived from exponential functions that were fitted to the data using the equation SWA_*t*_ = (SWA_0_ * e^-*rt*^) + SWA_∞_, in which SWA_*t*_ is the SWA value at a given time point *t*, *SWA*_*0*_ represents the hypothetical SWA value at time 0, *t* indicates time (min) from sleep onset, *r* is the rate (min^-1^) of exponential decay, and *SWA*_∞_ indicates the SWA value of the decay function’s asymptote. *CI* denotes the 95% confidence interval. *R*^*2*^ for the exponential function reflects goodness of fit.

Although MDD and HC subjects were age and sex-matched in the unstratified analysis, when stratified by sex, the ages between male and female participants were not identical (Table 1). Therefore, a secondary analysis was performed using analysis of covariance (ANCOVA) to compare differences in all-night global SWA among all groups that included age as a covariate. ANCOVA revealed a significant main effect of sex (F_1,55_ = 14.00, p = 0.0004), a trend towards main effect of diagnosis (F_1,55_ = 3.24, p = 0.077), a significant diagnosis x sex interaction (F_1,55_ = 5.98, p = 0.018), and a significant effect of age (F_1,55_ = 4.99, p = 0.030). Adjusted means for SWA were marginally different compared to unadjusted values (adjusted mean ± std error: MDD women: 28.05 ± 1.94, MDD men: 13.56 ± 2.60, HC women: 18.55 ± 1.95, HC men: 15.00 ± 2.56 μV^2^/Hz). Post hoc analysis showed MDD women demonstrated significantly greater SWA than HC women (p = 0.0009), HC men (p = 0.0002), and MDD men (p < 0.0001). However, there were no other significant differences in SWA between groups including between HC women and HC men (p = 0.28).

There were no significant correlations of SWA (either all-night or in NREM1) with HRSD scores in MDD, either stratified or unstratified by sex. Additionally, there were no significant correlations of polysomnographic variables with global or topographic SWA (either all-night or NREM1) in HC or MDD subjects, either stratified or unstratified by sex.

## Discussion

Our findings demonstrated an increase in SWA in young to middle aged women with unipolar MDD. Moreover, increases in SWA in MDD women primarily occurred during the first portion of the night and were most prominent in prefrontal regions. In contrast, men with MDD did not demonstrate significant changes in SWA, corroborating prior investigations that demonstrate sex-specific differences in SWA in MDD [29,30]. Strengths of this investigation include age and sex-matching of subjects, which allowed for analyses stratified by sex, as well as lack of confounding psychotropic medications and use of hdEEG for spectral analysis. These results highlight the importance of both sex and EEG topography in the evaluation of SWA in mood disorders.

The findings of this study must be taken in context of the ratio of women to men, which was roughly 2:1, approximating the differential prevalence of MDD by sex in the general population [46]. Notably, two known prior studies in the literature that have demonstrated similarly increased SWA or delta counts (using period amplitude analysis) in depressive illness relative to controls have both had disproportionately high ratios of female to male MDD subjects of greater than 3:1; however, neither study stratified their analyses by sex [47,48]. Our stratified analysis found that in women with MDD, SWA was globally increased relative to female HC, but was most prominently increased in bilateral prefrontal cortical regions, consistent with multiple structural and functional neuroimaging investigations which have demonstrated the importance of the prefrontal cortex in the neurobiology of depression [49,50]. However, it is currently not clear how increased SWA in women with MDD is related to the pathophysiology of the disorder.

This study highlights the importance of electrode placement in the evaluation of SWA during sleep in mood disorders. Notably, we did not find a significant difference in SWA between MDD and control subjects in central channels, including analyses stratified by sex. Since the previous scientific literature regarding SWA in depression has utilized central EEG derivations, it is conceivable that the aforementioned inconsistencies between previous studies may be in part due to a lack of topographical resolution or increased variability of SWA in central regions. Thus, the findings of this study suggest spectral analysis of sleep using central derivations may be inadequate to capture the alterations in SWA occurring across the cortex in mood disorders.

There are limitations of this study that merit discussion. Although a prior investigation has demonstrated decreases in SWA in men with MDD [30], we did not find statistically significant differences in men with MDD relative to matched HC subjects. However, it is possible that our study was underpowered to detect differences in SWA or decline of SWA across sleep among males. It is also possible that significant prefrontal increases in SWA in MDD in the unstratified analysis would not have been demonstrated had the ratio of men to women been proportionate. Furthermore, subjects did not have an adaptation night in the sleep laboratory, which may have affected results, if groups were differentially affected by first-night effects. Although delta EEG activity is not significantly different in the luteal versus follicular phase of the menstrual cycle in either healthy subjects or patients with severe premenstrual syndrome, it is also possible that not assessing menstrual status at the time of polysomnography may have influenced the results [51]. Finally, subjects were young to middle aged, and thus, results may not be applicable to older subjects with MDD, as it has been suggested alterations in SWA in MDD are more apparent in young to middle aged cohorts [31].

This study was not able to determine whether increased SWA in MDD is a causal phenomenon for the disorder or an epiphenomenon resulting from chronic sleep disruption associated with the illness. Topographic increases in SWA in depressed women observed in this study are not specific to MDD, as such findings are also seen during recovery sleep following acute sleep deprivation [33-35]. It is intriguing that increases in SWA are observed in both MDD and sleep deprivation paradigms, as both are associated with impairments in cognitive functions such as attention and working memory, as well as subjective fatigue [52,53]. Given the results of this study, further experiments are warranted that examine the relationship between sleep homeostatic processes and executive function in MDD, using sleep restriction and/or sleep delay paradigms [28], and the effects of sex on these parameters.

Our results suggest that increased SWA, particularly during early portions of the night, may be related to the pathophysiology of MDD in women. In accordance with this hypothesis, it has been previously proposed that increases in NREM sleep intensity may be depressogenic [54], and a recent demonstration that selective slow-wave deprivation has an acute antidepressant response supports this contention [55]. In addition, when compared to healthy controls, young MDD women exhibited greater low-frequency EEG activity in frontal regions during extended wakefulness [56], which has been demonstrated to be a waking correlate of sleep homeostasis [34,57]. Both increased SWA during baseline sleep and an enhanced homeostatic response to sleep deprivation paradigms have also been reported, suggesting that MDD women live with an elevated level of homeostatic sleep pressure [31,58,59]. Although our results would support this contention, our experimental design did not include manipulations of sleep homeostasis, and thus only provides partial evidence for this hypothesis. Moreover, given our cross-sectional design, we were not able to determine whether increased SWA in MDD is a state or trait marker for MDD in women, nor are we able to determine if increased SWA is causative for depressive symptoms or an epiphenomenon of some other neurobiological process, which may be mediated by sex-related differences in neurobiology.

The differences in brain structure and function between men and women, and how these alterations may relate to the pathophysiology of mood disorders is a complex topic, as dissecting which components are biologically determined and which are the consequence of environmental conditioning due to gender-related sociocultural experience is fraught with difficulty [60]. Still, there is significant evidence that there are sex-related differences in gene expression, epigenetic regulatory mechanisms, hypothalamus-pituitary-adrenal axis regulation, and modulation of neurotransmitter systems by neurosteroids between men and women, which may relate to differences in susceptibility to neuropsychiatric illness [61-63]. It is currently not clear how our neurophysiologic finding of increased SWA in women with MDD may be related to any of these mechanisms, and thus, further research that examines SWA in relation to sex-dependent hormonal, genetic, and epigenetic mechanisms in mood disorders may provide deeper insights into this conceptual framework.

## Conclusions

In conclusion, sleep disturbance and MDD are intimately linked; however, previous research that has examined the role of SWA in depression has been inconsistent. This study demonstrates sex-related differences in SWA, in which women with MDD had elevated SWA, most prominently in prefrontal regions and during the first portion of the night. Failure to account for sex-related differences in SWA and the use of limited EEG derivations to perform spectral analysis in prior investigations may have contributed to inconsistencies in the literature. However, given the heterogeneity of MDD [50,64], it is also plausible that other factors contribute to alterations in SWA in the disorder. Further research is indicated to examine the effects of sex-specific neurobiology, neurocognitive symptoms, and SWA in mood disorders to clarify how increased SWA in women with MDD is related to the pathophysiology of the disorder.

## Competing interests

DTP has owned stock in Pfizer, and has received honoraria from Oakstone Medical Publishing and royalties from Cambridge University Press. MJP has received unrelated research support from Sanofi-Aventis. GT has consulted for Sanofi-Aventis and Takeda, and he is currently the David P. White Chair in Sleep Medicine at the University of Wisconsin–Madison, endowed by Phillips Respironics. GT has also received unrelated research support from Phillips Respironics. RMB has consulted for Merck and Sanofi-Aventis. All other authors declare they have no competing interests.

## Authors’ contributions

DTP designed the study, managed literature searches and analyses, and wrote the initial and final draft of the manuscript. ECL and MJP contributed to the study design, evaluation of participants, management of hdEEG processing and analyses, and writing the article. MRG, TW, and JJG performed statistical analyses and hdEEG processing, designed tables/figures, and contributed to the writing of the manuscript. BA performed data analysis and interpretation, and also contributed to the writing of the manuscript. GT contributed to the study design, data analysis and interpretation, and to writing the article. RMB contributed by being the principal investigator, contributing to the study design, participating in data analysis and interpretation, and writing the article. All authors contributed to and have approved the final manuscript.

## Pre-publication history

The pre-publication history for this paper can be accessed here:

http://www.biomedcentral.com/1471-244X/12/146/prepub

## Supplementary Material

Additional file 1 **Figure S1.** Topographic SWA (1-4.5 Hz) during NREM1 in MDD subjects versus healthy controls, both unstratified and stratified by sex. T-values plotted for the comparisons between groups (2-tailed, unpaired *t*-test) at each channel. The minimum and maximum t-values for each map are plotted in white and black respectively, with the corresponding numeric range for color scale (upper left). Corresponding p-values plotted for each channel with white dots denoting channels with significant between-group differences following statistical non-parametric mapping with suprathreshold cluster tests to correct for multiple comparisons. (PNG 927 kb) Click here for file

## References

[B1] American Psychiatric Association Diagnostic and Statistical Manual of Mental Disorders: Fourth Edition, Text Revision ed 2000 American Psychiatric Association, Washington, D.C.

[B2] Nutt D Wilson S Paterson L Sleep disorders as core symptoms of depression Dialogues Clin Neurosci 2008 10 3 329 336 18979946 10.31887/DCNS.2008.10.3/dnutt PMC3181883

[B3] Breslau N Roth T Rosenthal L Andreski P Sleep disturbance and psychiatric disorders: a longitudinal epidemiological study of young adults Biol Psychiatry 1996 39 6 411 418 10.1016/0006-3223(95)00188-3 8679786

[B4] Chang PP Ford DE Mead LA Cooper-Patrick L Klag MJ Insomnia in young men and subsequent depression. The Johns Hopkins Precursors Study Am J Epidemiol 1997 146 2 105 114 10.1093/oxfordjournals.aje.a009241 9230772

[B5] Roberts RE Shema SJ Kaplan GA Strawbridge WJ Sleep complaints and depression in an aging cohort: A prospective perspective Am J Psychiatry 2000 157 1 81 88 10618017 10.1176/ajp.157.1.81

[B6] Buysse DJ Angst J Gamma A Ajdacic V Eich D Rossler W Prevalence, course, and comorbidity of insomnia and depression in young adults Sleep 2008 31 4 473 480 18457234 10.1093/sleep/31.4.473 PMC2279748

[B7] Szklo-Coxe M Young T Peppard PE Finn LA Benca RM Prospective associations of insomnia markers and symptoms with depression Am J Epidemiol 2010 171 6 709 720 10.1093/aje/kwp454 20167581 PMC2842222

[B8] Wojnar M Ilgen MA Wojnar J McCammon RJ Valenstein M Brower KJ Sleep problems and suicidality in the National Comorbidity Survey Replication J Psychiatr Res 2009 43 5 526 531 10.1016/j.jpsychires.2008.07.006 18778837 PMC2728888

[B9] Goldstein TR Bridge JA Brent DA Sleep disturbance preceding completed suicide in adolescents J Consult Clin Psychol 2008 76 1 84 91 18229986 10.1037/0022-006X.76.1.84 PMC2823295

[B10] Dombrovski AY Mulsant BH Houck PR Mazumdar S Lenze EJ Andreescu C Cyranowski JM Reynolds CF 3rd Residual symptoms and recurrence during maintenance treatment of late-life depression J Affect Disord 2007 103 1–3 77 82 17321595 10.1016/j.jad.2007.01.020 PMC2680091

[B11] Karp JF Buysse DJ Houck PR Cherry C Kupfer DJ Frank E Relationship of variability in residual symptoms with recurrence of major depressive disorder during maintenance treatment Am J Psychiatry 2004 161 10 1877 1884 10.1176/appi.ajp.161.10.1877 15465986

[B12] Paykel ES Ramana R Cooper Z Hayhurst H Kerr J Barocka A Residual symptoms after partial remission: an important outcome in depression Psychol Med 1995 25 6 1171 1180 10.1017/S0033291700033146 8637947

[B13] Benca RM Obermeyer WH Thisted RA Gillin JC Sleep and psychiatric disorders. A meta-analysis Arch Gen Psychiatry 1992 49 8 651 668 10.1001/archpsyc.1992.01820080059010 1386215

[B14] Steiger A Kimura M Wake and sleep EEG provide biomarkers in depression J Psychiatr Res 2010 44 4 242 252 10.1016/j.jpsychires.2009.08.013 19762038

[B15] Rechtschaffen A Kales A A Manual of Standardized Terminology Techniques and Scoring System for Sleep States of Human Subjects 1968 Brain information Service/Brain Research institute, University of California at Los Angeles

[B16] Iber C Ancoli-Israel S Chesson AL Quan SF American Academy of Sleep Medicine The AASM Manual for the Scoring of Sleep and Associated Events: Rules, Terminology, and Technical Specifications 2007 First edition American Academy of Sleep Medicine, Westchester, Illinois

[B17] Borbely AA Baumann F Brandeis D Strauch I Lehmann D Sleep deprivation: effect on sleep stages and EEG power density in man Electroencephalogr Clin Neurophysiol 1981 51 5 483 495 10.1016/0013-4694(81)90225-X 6165548

[B18] Dijk DJ Regulation and functional correlates of slow wave sleep J Clin Sleep Med 2009 5 2 Suppl S6 S15 19998869 PMC2824213

[B19] Borbely AA Wirz-Justice A Sleep, sleep deprivation and depression. A hypothesis derived from a model of sleep regulation Hum Neurobiol 1982 1 3 205 210 7185793

[B20] Achermann P Dijk DJ Brunner DP Borbely AA A model of human sleep homeostasis based on EEG slow-wave activity: quantitative comparison of data and simulations Brain Res Bull 1993 31 1–2 97 113 8453498 10.1016/0361-9230(93)90016-5

[B21] Borbely AA A two process model of sleep regulation Hum Neurobiol 1982 1 3 195 204 7185792

[B22] Borbely AA The S-deficiency hypothesis of depression and the two-process model of sleep regulation Pharmacopsychiatry 1987 20 1 23 29 10.1055/s-2007-1017069 3823126

[B23] Borbely AA Tobler I Loepfe M Kupfer DJ Ulrich RF Grochocinski V Doman J Matthews G All-night spectral analysis of the sleep EEG in untreated depressives and normal controls Psychiatry Res 1984 12 1 27 33 10.1016/0165-1781(84)90135-5 6589657

[B24] Hoffmann R Hendrickse W Rush AJ Armitage R Slow-wave activity during non-REM sleep in men with schizophrenia and major depressive disorders Psychiatry Res 2000 95 3 215 225 10.1016/S0165-1781(00)00181-5 10974360

[B25] Mendelson WB Sack DA James SP Martin JV Wagner R Garnett D Milton J Wehr TA Frequency analysis of the sleep EEG in depression Psychiatry Res 1987 21 2 89 94 10.1016/0165-1781(87)90067-9 3615694

[B26] Armitage R Calhoun JS Rush AJ Roffwarg HP Comparison of the delta EEG in the first and second non-REM periods in depressed adults and normal controls Psychiatry Res 1992 41 1 65 72 10.1016/0165-1781(92)90019-Y 1561289

[B27] Landolt HP Gillin JC Similar sleep EEG topography in middle-aged depressed patients and healthy controls Sleep 2005 28 2 239 247 16171249 10.1093/sleep/28.2.239

[B28] Brower KJ Hoffmann R Conroy DA Arnedt JT Armitage R Sleep homeostasis in alcohol-dependent, depressed and healthy control men Eur Arch Psychiatry Clin Neurosci 2011 261 8 559 566 10.1007/s00406-011-0195-5 21312040 PMC3156901

[B29] Armitage R Hoffmann R Fitch T Trivedi M Rush AJ Temporal characteristics of delta activity during NREM sleep in depressed outpatients and healthy adults: group and sex effects Sleep 2000 23 5 607 617 10947028

[B30] Armitage R Hoffmann R Trivedi M Rush AJ Slow-wave activity in NREM sleep: sex and age effects in depressed outpatients and healthy controls Psychiatry Res 2000 95 3 201 213 10.1016/S0165-1781(00)00178-5 10974359

[B31] Armitage R Sleep and circadian rhythms in mood disorders Acta Psychiatr Scand Suppl 2007 433 104 115 17280576 10.1111/j.1600-0447.2007.00968.x

[B32] Werth E Achermann P Borbely AA Fronto-occipital EEG power gradients in human sleep J Sleep Res 1997 6 2 102 112 10.1046/j.1365-2869.1997.d01-36.x 9377529

[B33] Cajochen C Foy R Dijk DJ Frontal predominance of a relative increase in sleep delta and theta EEG activity after sleep loss in humans Sleep Res Online 1999 2 3 65 69 11382884

[B34] Finelli LA Baumann H Borbely AA Achermann P Dual electroencephalogram markers of human sleep homeostasis: correlation between theta activity in waking and slow-wave activity in sleep Neuroscience 2000 101 3 523 529 10.1016/S0306-4522(00)00409-7 11113301

[B35] Tinguely G Finelli LA Landolt HP Borbely AA Achermann P Functional EEG topography in sleep and waking: state-dependent and state-independent features NeuroImage 2006 32 1 283 292 10.1016/j.neuroimage.2006.03.017 16650779

[B36] First M Spitzer R Gibbon M Williams J Structured clinical interview for DSM-IV-TR axis I disorders, research version, patient edition 2002 Biometrics Research, New York State Psychiatric Institute, New York

[B37] Hamilton M A rating scale for depression J Neurol Neurosurg Psychiatry 1960 23 56 62 10.1136/jnnp.23.1.56 14399272 PMC495331

[B38] First M Spitzer R Gibbon M Williams J Structured clinical interview for DSM-IV-TR axis I disorders, research version, non-patient edition (SCID-I/NP) 2002 Biometrics Research, New York State Psychiatric Institute, New York

[B39] Goncharova II McFarland DJ Vaughan TM Wolpaw JR EMG contamination of EEG: spectral and topographical characteristics Clin Neurophysiol 2003 114 9 1580 1593 10.1016/S1388-2457(03)00093-2 12948787

[B40] Dijk DJ Brunner DP Borbély AA Time course of EEG power density during long sleep in humans Am J Physiol 1990 258 3Pt2 R650 R661 2316712 10.1152/ajpregu.1990.258.3.R650

[B41] Feinburg I Floyd TC Systematic trends across the night in human sleep cycles Psychophysiology 1979 16 3 283 291 10.1111/j.1469-8986.1979.tb02991.x 220659

[B42] Mourtazaev MS Kemp B Zwinderman AH Kamphuisen HA Age and gender affect different characteristics of slow waves in the sleep EEG Sleep 1995 18 7 557 564 8552926 10.1093/sleep/18.7.557

[B43] Ehlers CL Kupfer DJ Slow-wave sleep: do young adult men and women age differently? J Sleep Res 1997 6 3 211 215 10.1046/j.1365-2869.1997.00041.x 9358400

[B44] Latta F Leproult R Tasali E Hofmann E Van Cauter E Sex differences in delta and alpha EEG activities in healthy older adults Sleep 2005 28 12 1525 1534 16408411 10.1093/sleep/28.12.1525

[B45] Nichols TE Holmes AP Nonparametric permutation tests for functional neuroimaging: a primer with examples Hum Brain Mapp 2002 15 1 1 25 10.1002/hbm.1058 11747097 PMC6871862

[B46] Kessler RC McGonagle KA Swartz M Blazer DG Nelson CB Sex and depression in the National Comorbidity Survey. I: Lifetime prevalence, chronicity and recurrence J Affect Disord 1993 29 2–3 85 96 8300981 10.1016/0165-0327(93)90026-g

[B47] Reynolds CF 3rd Kupfer DJ Taska LS Hoch CH Sewitch DE Grochocinski VJ Slow wave sleep in elderly depressed, demented, and healthy subjects Sleep 1985 8 2 155 159 4012158 10.1093/sleep/8.2.155

[B48] Schwartz PJ Rosenthal NE Wehr TA Band-specific electroencephalogram and brain cooling abnormalities during NREM sleep in patients with winter depression Biol Psychiatry 2001 50 8 627 632 10.1016/S0006-3223(01)01097-6 11690599

[B49] Drevets WC Price JL Furey ML Brain structural and functional abnormalities in mood disorders: implications for neurocircuitry models of depression Brain Struct Funct 2008 213 1–2 93 118 18704495 10.1007/s00429-008-0189-x PMC2522333

[B50] Krishnan V Nestler EJ Linking molecules to mood: new insight into the biology of depression Am J Psychiatry 2010 167 11 1305 1320 10.1176/appi.ajp.2009.10030434 20843874 PMC3031089

[B51] Baker FC Sassoon SA Kahan T Palaniappan L Nicholas CL Trinder J Colrain IM Perceived poor sleep quality in the absence of polysomnographic sleep disturbance in women with severe premenatrual syndrome J Sleep Res In Press 10.1111/j.1365-2869.2012.01007.x PMC3376683 22417163

[B52] Marazziti D Consoli G Picchetti M Carlini M Faravelli L Cognitive impairment in major depression Eur J Pharmacol 2010 626 1 83 86 10.1016/j.ejphar.2009.08.046 19835870

[B53] Goel N Rao H Durmer JS Dinges DF Neurocognitive consequences of sleep deprivation Semin Neurol 2009 29 4 320 339 10.1055/s-0029-1237117 19742409 PMC3564638

[B54] Beersma DG van den Hoofdakker RH Can non-REM sleep be depressogenic? J Affect Disord 1992 24 2 101 108 10.1016/0165-0327(92)90024-Z 1541764

[B55] Landsness EC Goldstein MR Peterson MJ Tononi G Benca RM Antidepressant effects of selective slow wave sleep deprivation in major depression: a high-density EEG investigation J Psychiatr Res 2011 45 8 1019 1026 10.1016/j.jpsychires.2011.02.003 21397252 PMC3119746

[B56] Birchler-Pedross A Frey S Chellappa SL Gotz T Knoblauch V Wirz-Justice A Cajochen C Higher frontal EEG synchronisation in young women with major depression: a marker for increased homeostatic sleep pressure? Sleep 2011 34 12 1699 1706 22131608 10.5665/sleep.1440 PMC3208848

[B57] Cajochen C Wyatt JK Czeisler CA Dijk DJ Separation of circadian and wake duration-dependent modulation of EEG activation during wakefulness Neuroscience 2002 114 4 1047 1060 10.1016/S0306-4522(02)00209-9 12379258

[B58] Birchler-Pedross A Frey S Chellappa SL Götz T Brunner P Knoblauch V Wirz-Justice A Cajochen C Higher frontal EEG synchronization in young women with major depression: a marker for increased homeostatic sleep pressure? Sleep 2011 34 12 1699 706 22131608 10.5665/sleep.1440 PMC3208848

[B59] Frey S Birchler-Pedross A Hofstetter M Brunner P Götz T Münch M Blatter K Knoblauch V Wirz-Justice A Cajochen C Young women with major depression live on higher homeostatic sleep pressure than healthy controls Chronobiol Int 2012 29 3 278 294 10.3109/07420528.2012.656163 22390241

[B60] Legato MJ The skewed sex distribution in affective disorders–a diagnostic, social, or biological problem? Prog Brain Res 2010 186 159 166 21094891 10.1016/B978-0-444-53630-3.00010-5

[B61] Qureshi IA Mehler MF Genetic and epigenetic underpinnings of sex differences in the brain and in neurological and psychiatric disease susceptibility Prog Brain Res 2010 186 77 95 21094887 10.1016/B978-0-444-53630-3.00006-3 PMC4465286

[B62] Reddy DS Neurosteroids: endogenous role in the human brain and therapeutic potentials Prog Brain Res 2010 186 113 137 21094889 10.1016/B978-0-444-53630-3.00008-7 PMC3139029

[B63] Young E Korszun A Sex, trauma, stress hormones and depression Mol Psychiatry 2010 15 1 23 28 10.1038/mp.2009.94 19773810

[B64] Tsuno N Besset A Ritchie K Sleep and depression J Clin Psychiatry 2005 66 10 1254 1269 10.4088/JCP.v66n1008 16259539

