# Supplementary figures and images for: Sex-related differences in sleep slow wave activity in major depressive disorder: a high-density EEG investigation
Source: BMC Psychiatry. 2012 Sep 18;12:146. doi: 10.1186/1471-244X-12-146 (PMC3507703; doi:10.1186/1471-244X-12-146)

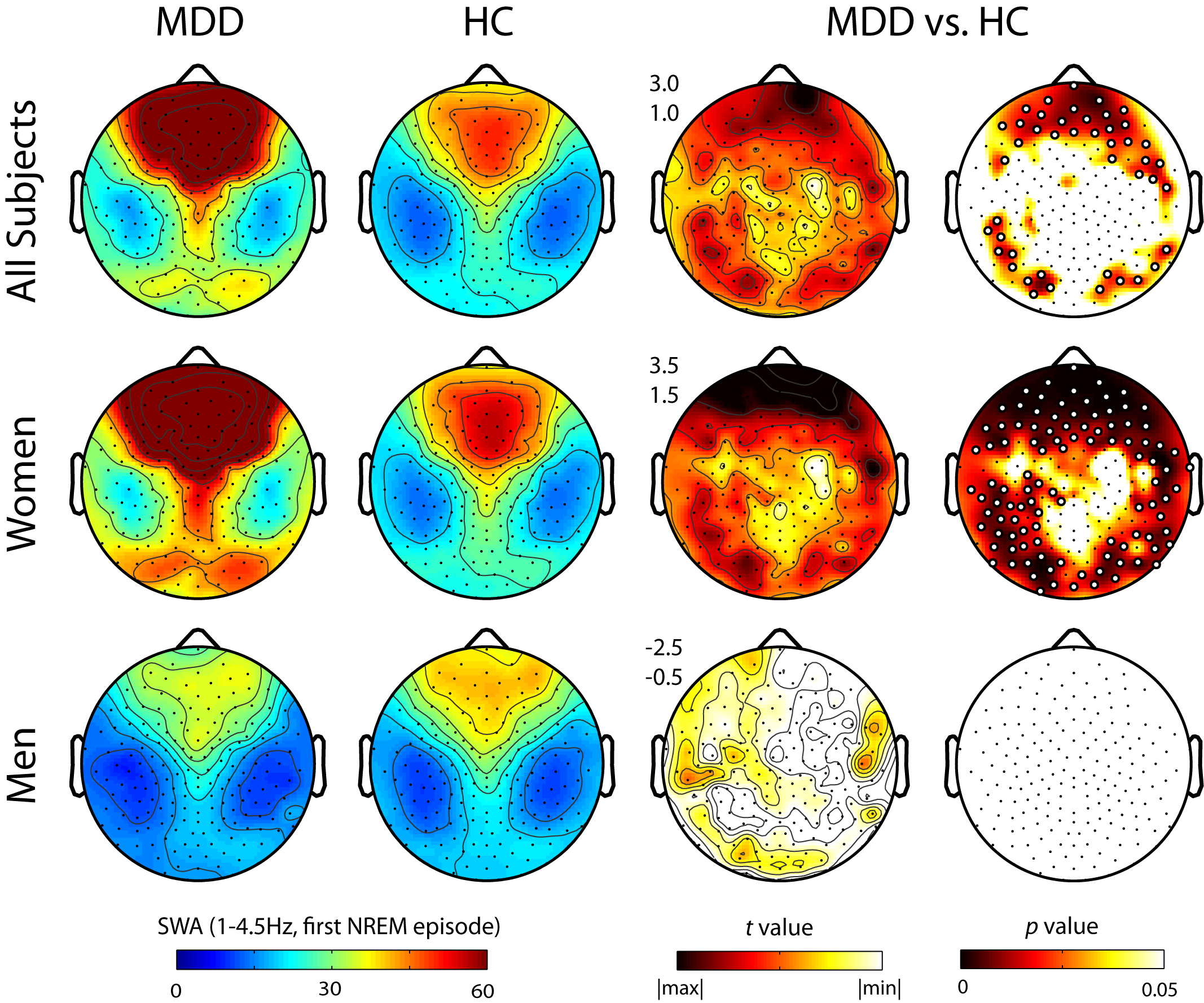

Supplement: Additional file 1 — Figure S1. Topographic SWA (1-4.5 Hz) during NREM1 in MDD subjects versus healthy controls, both unstratified and stratified by sex. T-values plotted for the comparisons between groups (2-tailed, unpaired t-test) at each channel. The minimum and maximum t-values for each map are plotted in white and black respectively, with the corresponding numeric range for color scale (upper left). Corresponding p-values plotted for each channel with white dots denoting channels with significant between-group differences following statistical non-parametric mapping with suprathreshold cluster tests to correct for multiple comparisons. (PNG 927 kb) [file 1471-244X-12-146-S1.png]
